# Effects of Cigarette Smoking on Transplant Survival: Extending or Shortening It?

**DOI:** 10.3389/fimmu.2017.00127

**Published:** 2017-02-10

**Authors:** Feifei Qiu, Ping Fan, Golay D. Nie, Huazhen Liu, Chun-Ling Liang, Wanlin Yu, Zhenhua Dai

**Affiliations:** ^1^Section of Immunology, Guangdong Provincial Academy of Chinese Medical Sciences, Guangzhou, China; ^2^Department of Nephrology, Shaanxi Provincial Hospital of Chinese Medicine, Xi’an, China; ^3^School of Medicine, University of Texas Medical Branch, Galveston, TX, USA

**Keywords:** cigarette smoking, adaptive immunity, innate immunity, allograft survival, transplant tolerance

## Abstract

Cigarette smoking (CS) regulates both innate and adaptive immunity and causes numerous diseases, including cardiovascular, respiratory, and autoimmune diseases, allergies, cancers, and transplant rejection. Therefore, smoking poses a serious challenge to the healthcare system worldwide. Epidemiological studies have always shown that CS is one of the major risk factors for transplant rejection, even though smoking plays redundant roles in regulating immune responses. The complex roles for smoking in immunoregulation are likely due to molecular and functional diversities of cigarette smoke components, including carbon monoxide (CO) and nicotine. Especially, CO has been shown to induce immune tolerance. Although CS has been shown to impact transplantation by causing complications and subsequent rejection, it is overlooked whether CS interferes with transplant tolerance. We have previously demonstrated that cigarette smoke exposure reverses long-term allograft survival induced by costimulatory blockade. Given that CS impacts both adaptive and innate immunity and that it hinders long-term transplant survival, our perspective is that CS impacts transplant tolerance. Here, we review impacts of CS on major immune cells that are critical for transplant outcomes and propose the cellular and molecular mechanisms underlying its effects on alloimmunity and transplant survival. Further investigations are warranted to fully understand why CS exerts deleterious rather than beneficial effects on transplant survival even if some of its components are immunosuppressive.

## Introduction

**Figure 1:**
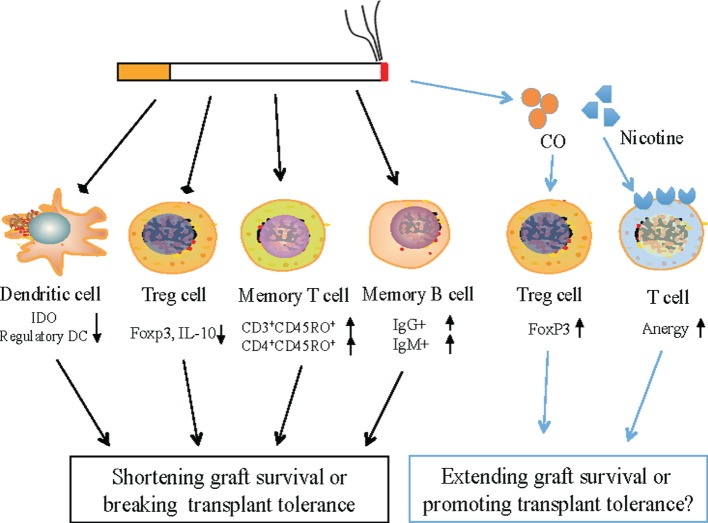
**Potential mechanisms by which cigarette smoking (CS) alters transplant rejection and interferes with transplant tolerance**. CS represses the expression of IDO in dendritic cells (DCs) and Foxp3 in regulatory T cells (Tregs) and hence reduces the percentages of regulatory DCs as well as FoxP3+ Tregs, but increases the frequency of CD4+ or CD3+ memory T cells and IgG+/IgM+ memory B cells, likely resulting in transplant rejection or the loss of immune tolerance as the balance between pathogenic memory T/B cells and regulatory DCs/Tregs is interrupted by smoking. However, as a cigarette smoke component, carbon monoxide generally induces Tregs while nicotine promotes T cell anergy. Therefore, both CS components could suppress allograft rejection if sufficient amounts were inhaled.

Cigarette smoking (CS) contributes to various diseases, including oral, respiratory, and cardiovascular diseases, infections, cancers, autoimmune diseases, and transplant rejection. It impacts both adaptive and innate immunity. On the other hand, CS is a well-known risk factor associated with the morbidity and mortality of organ transplantation. Numerous epidemiological analyses and retrospective reviews have shown that CS increases the risks of transplantation-related complications, allograft rejection, and recipient death in transplanted patients. Although the adverse impacts of smoking on outcomes of organ transplantation are well documented, the molecular and cellular mechanisms responsible for smoking-associated graft rejection are neither fully understood nor carefully proposed. Especially, it has been overlooked whether and how smoking directly impacts transplant tolerance, even with many of the original studies showing that CS has widely affected immune responses as well as transplantation outcomes. In the current review, we summarize the effects of smoking on the major immune cells that impact transplant survival or tolerance (Figure 1) and propose the molecular mechanisms that are likely responsible for CS-associated allograft rejection.

## Effects of Smoking on Transplant Survival

Growing studies have demonstrated that smoking hinders long-term allograft survival by promoting various complications after transplantation. Pungpapong et al. reported that patients with a smoking history had higher risks of vascular complications, especially arterial ones than non-smokers after liver transplantation, while patients, who gave up smoking for 2 years prior to transplantation, had lower incidence of vascular complications (1). Further studies revealed that CS was associated with increased incidence of biliary complication and higher hepatocellular carcinoma rates (2). Similarly, CS by both donors and recipients had negative impacts on outcomes of lung transplantation. Smoking by donors directly influenced the early postoperative events rather than the late outcomes (3). Studies on former and current smokers with lung transplantation demonstrated that smokers had higher incidence of decreased pulmonary function or chronic kidney disease, a common complication of lung transplantation, than non-smoking recipients (4). Moreover, pretransplantation smoking by either donors or recipients significantly reduced recipient survival rates (5). CS after renal transplantation was associated with impaired renal function (6), vascular intimal fibrous thickening, and higher incidence of diabetes (7). Adverse effects of smoking on heart transplantation were also reported. Nägele et al. found that smokers with heart transplantation had less long-term survivors and higher incidence of transplant vasculopathy than non-smokers (8). Pretransplantation and posttransplantation smoking not only resulted in a poorer prognosis and a longer period of recovery but also significantly raised overall mortality of patients (9–11).

Although numerous epidemiological studies supported the finding that CS is an important risk factor for organ transplantation, there was a lack of the proof that smoking can directly cause allograft failure in a cause-effect manner. Recently, our studies have provided the first evidence that second-hand smoking (SHS) directly prevents long-term allograft survival, which would otherwise have been induced by the costimulatory blockade in animal models (12). We found that SHS dramatically reduced the intragraft expression of indoleamine2,3-dioxygenase (IDO) while overexpression of IDO by islet allografts resumed long-term islet allograft survival induced by the costimulatory blockade even in the face of SHS. Thus, we demonstrated that smoke exposure directly facilitated allograft rejection and interrupted transplant tolerance by repressing the expression and activity of IDO, which would otherwise have suppressed T cell activation and facilitated the immune tolerance (13).

## Impacts of Smoking on Major Immune Cells That Mediate Transplant Rejection or Promote Graft Survival

It is well known that allograft rejection can be suppressed or prevented by either global immunosuppression or targeted immunomodulation. The balanced activation of conventional dendritic cells (DCs), T cells and B cells, regulatory T cells (Treg) and regulatory B cells, and memory T and B cells is vital for the appropriate regulation of immunity, which determines transplantation outcomes. Since CS impacts both innate and adaptive immunity, it likely also affects immune-based transplant rejection or allograft tolerance (Figure 1).

## Dendritic Cells

Dendritic cells, a critical subset of innate immune cells that serve as antigen-presenting cells, play an important role in mediating T cell activation as well as regulation of T cell function. Oberhuber et al. demonstrated that DCs expressing CD11c in murine recipients facilitated rejection of cardiac allograft and promoted the proliferation of both CD4+ and CD8+ T cells (14). Furthermore, analyses of renal transplant biopsies from patients indicated that high density of DCs was associated with increased proliferation of T cells and the poor outcomes (15). On the other hand, increasing evidence also showed that some subsets of DCs were immunosuppressive and could induce tolerance (16). Therefore, DCs play dual roles in alloimmune responses. DCs with immunoregulatory function were also known as tolerogenic DCs (Tol-DCs) as IDO-expressing Tol-DCs suppressed allograft rejection and prolonged recipient survival after small bowel and cardiac transplantation (17). These studies suggest that DCs mediate allograft rejection while IDO-expressing DCs or Tol-DCs are capable of suppressing allograft rejection or inducing transplant tolerance.

It has been reported that CS interferes with DC function. Previous studies on airway inflammation have shown that CS increases the number of DCs (18), promotes the trafficking and function of DCs (19, 20) and, therefore, induces allergic airway inflammation. We have previously found that SHS suppresses intragraft IDO expression and reverses long-term allograft survival induced by costimulatory blockade in a murine model (12). Since IDO is mainly expressed by DCs, these studies indicate that smoking is likely to aggravate transplantation rejection by either stimulating the frequency and function of DCs or repressing their IDO expression. However, other studies have shown that CS is associated with impaired DC maturation and lower capacity of DCs to stimulate T cell proliferation (21). Le Rouzic et al. found that CS exposure was associated with an inhibition of DC capacity to activate antigen-specific T-cell response, resulting in defective pro-Th1 and -Th17 responses to bacteria in chronic obstructive pulmonary disease (COPD) patients (22). These studies demonstrated that CS weakened immunity. Therefore, CS likely exerts dual effects on DCs. It remains to be defined how CS influences allograft rejection by altering DC function.

## CD4+ CD25+ Tregs

It is well known that Tregs play a key role in maintaining the immunologic self-tolerance and regulating immune responses to exogenous antigens in case of excessive immune reactions. Alterations in either functionality or number of Tregs may result in autoimmune diseases or loss of transplantation tolerance. Transfer of CD4+ CD25+ Tregs not only suppresses allograft rejection and graft-versus-host disease but also promotes transplant tolerance induction. While it is unclear whether CS alters Treg numbers and function in the context of transplantation, previous studies have revealed a significant downregulation of CD4+ CD25+ Treg cell numbers in bronchoalveolar lavage fluid and blood of smoking patients with COPD compared with healthy non-smokers (23, 24). Since our previous studies have demonstrated that second-hand smoke reduces the expression and activity of IDO in an allograft (12), it is likely that smoking also inhibits Treg generation in the context of transplantation, because IDO generally promotes Treg development and activation (25). It is also possible that CS suppresses the generation of Tregs or reduces their stability via increasing pro-inflammatory cytokine IL-6 (26), which has been shown to destabilize FoxP3 expression.

## Memory T Cells

Memory T cells can rapidly initiate alloimmune responses through the production of pro-inflammatory or effector cytokines (27). Previous studies also have revealed that trafficking limitation of memory CD4+ T cells by sphingosine 1-phosphate receptor-1 agonist delays transplant rejection and improves survival of cardiac allografts (28). Similarly, depletion of CD8+ memory T cells using an anti-CD8 antibody was reported to be an effective approach to inducing allograft tolerance in non-human primates that have undergone kidney transplantation (29). Acute rejection of a liver transplant in patients was associated with proliferation of CD8+ memory T cells infiltrating the allograft and with their increased expression of IFN-γ and IL-17 (30). Hence, memory T cells were considered to be a major barrier to long-term allograft survival or transplant tolerance (31). As mounting evidence has shown that either active or passive CS significantly increases the numbers of memory CD4+ T cells (32, 33), we propose that smoking stimulates cross-reactive memory T cells, which in turn accelerate allograft rejection or at least interfere with transplant tolerance induction.

## Memory B Cells

It is well accepted that both memory T and memory B cells prevent the long-term survival of transplanted organs and hinder the induction of transplant tolerance. Thus, much attention has been paid to the actual effects of memory B cells and alloantibodies on transplant rejection. Using a murine model of cardiac transplantation, Burns et al. found that allograft survival induced by costimulatory blockade was prevented by memory B cells and alloantibodies of recipients (34), while alloantibodies hindered transplant tolerance induction through priming alloreactive T cells (35). Given that memory B cells reportedly posed a long-term threat to allograft survival and that CS increased class-switched memory B cells in peripheral blood of current smokers and IgG+ memory B cells in the lung of COPD patients (36, 37), we propose that CS prevents allograft tolerance induction via enhancing either cross-reactive or allospecific B cell memory.

## Effects of Chemical Components of Cigarette Smoke on Immunity or Immune Tolerance

Cigarette smoke contains large amounts of toxic components, including carbon monoxide (CO), nicotine, nitrogen oxides, and cadmium. These harmful toxins alter the immune homeostasis in oral or airway mucosa and solid organs, resulting in regional inflammation and abnormal immune responses to exogenous antigens. However, emerging evidence has shown that CO and nicotine, two of the major cigarette smoke components, are generally immunosuppressive. Therefore, they could suppress allograft rejection or promote transplant tolerance induction (Figure 1).

## Carbon Monoxide

Carbon monoxide is produced when the heme is catalyzed by the enzyme heme oxygenase-1 (HO-1) expressed in immune cells (38), and it has been shown to exert immunosuppressive and antiapoptotic effects in animal studies. CO also suppresses alloimmune responses and allograft rejection. It was reported that either gene transfer of HO-1 or delivery of CO suppressed chronic rejection of cardiac xenografts (39). CO treatments prolonged survival of islet allografts, decreased expression of some pro-inflammatory and pro-apoptotic genes, and reduced macrophage infiltration in the grafts (40). Interestingly, CO was capable of maintaining peripheral tolerance by inducing CD4+ CD25+ Tregs (41). Therefore, either enhancing HO-1 expression or directly delivering CO ameliorated ischemia/reperfusion injury and graft rejection, alleviated chronic allograft failure, and facilitated donor-specific allograft tolerance (39, 42, 43).

However, it is unclear why smoking, which also produces CO, has not been found to prolong allograft survival. Instead, it is well accepted that smoking generally promotes transplant rejection and even hinders the induction of long-term transplant survival or tolerance. Perhaps, CS itself does not provide transplanted smokers a significant amount of CO that is sufficient for suppressing vigorous alloimmunity. It is also possible that smoking generates a mixture of hazardous materials that cooperate to hinder long-term allograft survival. In other words, beneficial effects of CO on allograft survival may be well offset by other harmful substances contained in cigarette smoke. Therefore, how smoking-generated CO exerts its effects on alloimmunity and allograft survival in smokers remains unclear and deserves further investigations.

## Nicotine

Similarly, an immunosuppressive feature of nicotine, another major component of cigarette smoke, was reported in several animal studies *in vitro* and *in vivo* (44, 45). For instance, nicotine suppressed lung Th2 responses (44), DC activation, and host responses to vaccination (45). Importantly, continuous nicotine treatments also induced T cell anergy (46). Early studies using animals demonstrated that nicotine promoted the revascularization of an autograft in the anterior chamber of rat eyes after bone transplantation (47). Studies using pulmonary transplant models also revealed that nicotine treatments significantly reduced the infiltration of CD68+ macrophage-like cells within pulmonary allografts and decreased the production of inducible NO synthase in alveolar macrophages (48), indicating that nicotine possibly ameliorates acute transplant rejection. On the other hand, Nordman et al. found that nicotine increased CD3+ CD4+ T cell numbers in circulation through activation of α4 nicotinic acetylcholine receptor (α4 nAChR) on T cells and especially promoted a Th2 response (49), which could affect transplant survival. Therefore, it remains unknown how nicotine alters immune-based rejection of a solid allograft, although it has been shown to be mostly immunosuppressive. However, nicotine could promote transplant tolerance induction since it was shown to induce T cell anergy. Further studies are warranted to determine whether and how nicotine regulates alloimmune responses and alters transplantation outcomes.

## The Molecular Mechanisms Underlying Smoking-Associated Immunopathology

**Figure 2:**
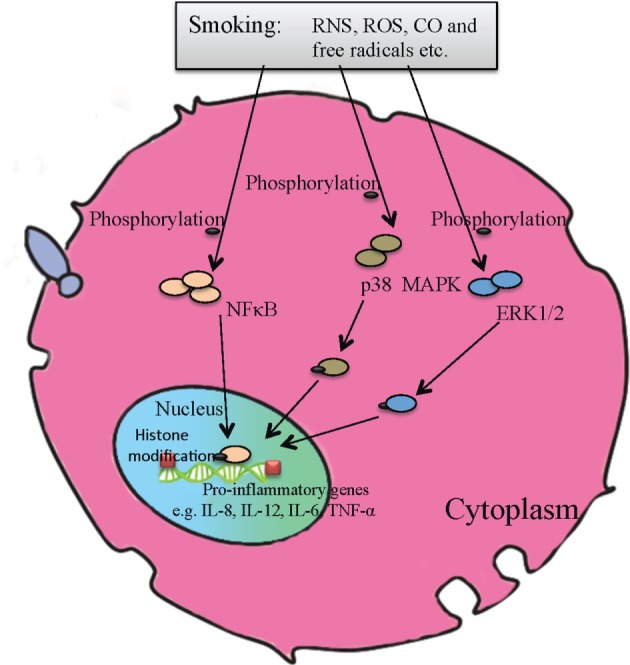
**A molecular model of pro-inflammatory signaling pathways activated by cigarette smoking**. Combustion products of cigarette smoke, including free radicals, carbon monoxide, reactive oxygen species, and reactive nitrogen species, can phosphorylate p38 mitogen-activated protein kinase and ERK1/2 signaling pathways, activate NFκB, and alter histone modification, resulting in aberrant expression of pro-inflammatory genes, subsequent inflammation and DNA damage, etc.

To further understand the effects of CS on alloimmunity and allograft rejection, it is important to know the molecular mechanisms by which smoking acts on the immune cells. Apart from CO and nicotine, cigarette smoke contains considerable adverse chemicals, including reactive nitrogen species (RNS), reactive oxygen species (ROS), and free radicals. These biohazards contribute to inflammation, oxidative stress, and DNA damage. On the other hand, toll-like receptors (TLRs) expressed in innate immune cells are critical for recognizing exogenous pathogens. Previous studies presented the evidence that CS triggered the acute inflammation via TLR4/MYD88 signaling pathway (50). It was also shown that smoking altered the expression profile of pro-inflammatory cytokines and chemokines through regulating signaling pathways and activities of some transcription factors. NFκB activation induced by oxidative stress from the smoke increased cyclooxygenase-2 and IL-8 expression (51, 52). Cigarette smoke extracts were also reported to promote phosphorylation of ERK1/2 and p38 mitogen-activated protein kinase (MAPK) and activation of their downstream pathways (Figure 2), leading to altered expression of heme oxygenase-1 (HO-1), IL-12, and IL-23 as well as IL-8 (53–55). Recently, it was demonstrated that ATP-P2X7-inflammasome-caspase 1-IL-1/18 axis played a role in smoke-induced airway inflammation in healthy smokers or COPD patients as well as murine COPD models, with increased activation of P2X7 receptor/caspase 1 and elevated IL-1β and IL-18 levels (56). In that study, it was found that the inflammasome-associated proteins ASC and NALP3 were required for IL-1β and IL-18 production induced by smoking (57). Furthermore, smoking exerted its influence on chromatin structures via mediating histone modification in pro-inflammatory macrophages (58), possibly resulting in abnormal gene transcriptions. Finally, Maldifassi et al. found that nicotine, through stimulation of α7 nicotinic acetylcholine receptor (α7 nAChR), played an anti-inflammatory role in human macrophages by upregulating the expression of interleukin-1 receptor-associated kinase M (IRAK-M), a negative regulator of innate TLR-mediated immune responses (59). They also provided evidence that activation of STAT3, through a single (JAK2/PI3K/STAT3) or two convergent cascades (JAK2/STAT3 and PI3K/STAT3), is required for nicotine-induced upregulation of IRAK-M expression (59).

Taken together, the signaling pathways and molecular mechanisms underlying CS-mediated immunopathology, especially transplant rejection, are still not well understood due to the functional complexity and diversity of cigarette smoke components. Further investigations are warranted to understand the molecular mechanisms of their action, especially those involved in alloimmune responses.

## Concluding Remark/Opinion

Smoking plays dual roles in immune responsiveness due to the molecular and functional diversities of cigarette smoke components, including RNS, ROS, CO, and nicotine. However, numerous experimental and clinical studies have shown that CS exerts a deleterious rather than beneficial effect on transplant survival even if CS downregulates immune responses under certain circumstances, for instance, smoking suppresses the immune system by reducing lymphocyte numbers in the blood of patients with chronic renal failure (60). Therefore, our view is that smoking hinders long-term allograft survival by stimulating T or B cell memory, suppressing Tol-DC development, or downregulating Treg cell numbers. Although combustion of cigarette produces CO and nicotine that are immunosuppressive, we think that the amount of CO and nicotine inhaled during smoking may not be sufficient to suppress vigorous allograft rejection. While previous studies have revealed some molecular mechanisms by which cigarette smoke alters immune responsiveness and mediates immunopathology, including P2X7-inflammasome-caspase 1-IL-1/18 axis, phosphorylation of ERK1/2 and p38 MAPK, and activation of NFκB in immune cells, the exact molecular mechanisms underlying smoking-associated transplant rejection remain largely unknown. Further studies are warranted to understand why smoking accelerates allograft rejection even if cigarette smoke components contain immunosuppressive substances, such as CO and nicotine, which would otherwise induce CD4+ CD25+ Tregs, promote T cell anergy, or suppress alloimmune responses. Hence, it is possible that the amount of CO or nicotine absorbed by smokers during smoking is insufficient to suppress allograft rejection. There is also a possibility that the inflammation and oxidative stress induced by other components of CS, such as RNS, ROS, and free radicals, can largely outweigh any potential immunosuppressive action of CO and nicotine.

## Author Contributions

FQ wrote the majority of the manuscript; PF and HL wrote a part of the manuscript; C-LL and WY prepared the literature; GN and ZD edited the manuscript.

## Conflict of Interest Statement

The authors declare that the research was conducted in the absence of any commercial or financial relationships that could be construed as a potential conflict of interest.
